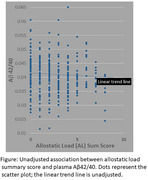# Association of Allostatic Load with Plasma Aβ 42/40 Ratio in Midlife: Evidence from the Child Health and Development Studies

**DOI:** 10.1002/alz70860_107371

**Published:** 2025-12-23

**Authors:** Pam Factor‐Litvak, Piera Cirillo, Isha Mhatre‐Winters, Carolyn Accardi, Young‐Mi Go, Dean P Jones, Nickilou Krigbaum, Katrina L Kezios, Barbara Cohn, Jason R Richardson

**Affiliations:** ^1^ Columbia University Mailman School of Public Health, New York, NY, USA; ^2^ Child Health and Development Studies, Public Health Institute, Oakland, CA, USA; ^3^ Isakson Center for Neurological Disease Research, College of Veterinary Medicine, University of Georgia, Athens, GA, USA; ^4^ Emory University School of Medicine, Atlanta, GA, USA

## Abstract

**Background:**

Allostatic load (AL) is a construct reflecting the accumulation of stressors and resulting in dysregulation of metabolic, inflammatory, cardiovascular and neuroendocrine systems over the life course. Studies examining associations between AL or its components and Alzheimer's Disease and Related Dimensia (ADRD), use cognitive tests, self‐reported status or white matter hyperintensity volumes as outcomes. Only a few use ADRD biomarkers, such as plasma Aβ 42/40 ratio and those derive their participants from specialty memory clinics. Here we examine associations between AL, its components and plasma Aβ 42/40 ratio in a longitudinal birth cohort.

**Method:**

Offspring born into the Child Health and Development Studies pregnancy cohort from 1960 ‐1963 in Oakland, CA were recruited for a follow‐up study of health disparities in 2010. Participants (*N* = 367) completed cognitive function assessments, were interviewed and provided blood samples, in their late 40's to early 50's. Aβ 42/40 was measured from stored plasma taken in midlife using the Quanterix N3PA kit. AL was assessed using 10 biomarkers reflecting metabolic, inflammatory, cardiovascular and neuroendocrine systems and was calculated as the number of components in the high risk quartiles. Associations were estimated using binomial logistic regression, where the outcome, low Aβ 42/40 was defined as the lowest tertile compared to the higher two tertiles. Models were adjusted for sex, race and educational attainment.

**Result:**

Higher AL was associated with an increased risk of low Aβ 42/40 (estimated odds ratio = 1.20; 95% confidence interval: 1.08, 1.34; *p* < .01) (Figure). High risk concentrations of biomarkers in the metabolic and inflammatory systems were individually associated with Aβ 42/40; one inflammatory and one neuroendocrine marker DHEA‐S were borderline associated with Aβ 42/40; cardiovascular markers were not associated. Results were robust to other definitions of allostatic load.

**Conclusion:**

In a sample of mid‐life adults without known memory problems, AL reflecting chronic stressors, is associated with plasma Aβ 42/40, a predictor of ADRD. As many of the biomarkers used to define AL are routinely used in health screening, early intervention may reduce the risk of ADRD later in life, but this needs to be confirmed in other studies.